# Neuronal p38 MAPK Signaling Contributes to Cisplatin-Induced Peripheral Neuropathy

**DOI:** 10.3390/antiox14040445

**Published:** 2025-04-08

**Authors:** Yugal Goel, Donovan A. Argueta, Kristen Peterson, Naomi Lomeli, Daniela A. Bota, Kalpna Gupta

**Affiliations:** 1 Hematology/Oncology, Department of Medicine, University of California, Irvine, CA 92697, USA; ygoel@hs.uci.edu (Y.G.); daarguet@hs.uci.edu (D.A.A.); kristeap@hs.uci.edu (K.P.); 2 Department of Neurology, Department of Medicine, University of California, Irvine, CA 92697, USA; nrlomeli@hs.uci.edu (N.L.); dbota@hs.uci.edu (D.A.B.); 3 Chao Family Comprehensive Cancer Center, University of California, Irvine, CA 92697, USA; 4 Division of Hematology, Oncology and Transplantation, Department of Medicine, University of Minnesota, Minneapolis, MN 55455, USA

**Keywords:** cancer, cisplatin, CIPN, dorsal root ganglion, neuron, pain, p38 MAPK

## Abstract

This study investigates the role of p38 mitogen-activated protein kinase (MAPK) activation in dorsal root ganglion (DRG) neurons in the development and progression of chemotherapy-induced peripheral neuropathy (CIPN). This research evaluates whether inhibiting activation of p38 MAPK could reduce neuropathic outcomes in a transgenic breast cancer mouse model (C3TAg) and wild-type mice (FVB/N) treated with cisplatin. Cisplatin treatment stimulated p38 MAPK phosphorylation and nuclear translocation in DRG neurons. Neflamapimod, a specific inhibitor of p38 MAPK alpha (p38α), proven to be safe in clinical trials, inhibited neuronal cisplatin-induced p38 MAPK phosphorylation in vitro and in vivo. Neflamapimod also reduced cisplatin-induced oxidative stress, mitochondrial dysfunction, and cleaved caspase-3 expression in DRG neurons in vitro, protecting neuronal integrity and preventing axonal damage. Functionally, neflamapimod improved mechanical and musculoskeletal hyperalgesia, and cold sensitivity in cisplatin-treated mice, reversing neuropathic pain and neurotoxicity. This study identifies p38 MAPK activation as a critical driver of CIPN and highlights its potential as a therapeutic target for CIPN. Targeting p38 MAPK activation with neflamapimod offers a promising strategy to mitigate neurotoxicity and hyperalgesia without exacerbating cancer progression, positioning it as a novel intervention for CIPN.

## 1. Introduction

Cisplatin (cis-diamminedichloroplatinum) and other platinum-based chemotherapies are the mainstays of treatment for many cancers [1]. However, chemotherapy-induced peripheral neuropathy (CIPN) associated with cisplatin treatment remains a frequent, painful, and dose-limiting toxicity [1,2,3,4]. With survival increasing across multiple cancer types, the number of patients suffering from chronic pain related to chemotherapy-induced neurotoxicity is increasing [5]. Chemotherapy affects the peripheral nervous system (PNS) neurons and axons in the dorsal root ganglion (DRG) [6] with progressive worsening of CIPN that persists following discontinuation of chemotherapy [1,4]. Neuropathic symptoms are most common in the distal extremities and may produce electric, burning, or freezing sensations, as well as hypersensitivity to touch (allodynia) and/or hyperalgesia [4]. Despite extensive research on the mechanisms of CIPN, there remains a critical lack of safe and effective therapies.

Targeting molecular pathways implicated in neuronal dysfunction, such as p38 mitogen-activated protein kinase (MAPK), presents a promising yet understudied approach to mitigating CIPN [7]. Murine studies have demonstrated that damage to peripheral sensory neurons of the DRG contributes to CIPN [8,9,10,11]. Exposing DRG neurons to cisplatin causes mitochondrial dysfunction and oxidative stress, leading to axonal degeneration, a primary contributor to the development of neuropathic pain [12,13]. Oxidative stress is associated with activation of the p38 MAPK signaling pathway in cisplatin-induced renal toxicity and peripheral neuropathy [14,15]. DRG neurons have thus been identified as potential targets for therapeutic interventions for CIPN [16].

We examined the potential of inhibiting p38 MAPK activity to ameliorate CIPN using neflamapimod (VX-745), an orally administered, highly specific, brain-penetrant, intracellular inhibitor of p38α MAPK [17]. Neflamapimod is a selective inhibitor of p38α and p38β MAPKs, which play critical roles in cellular stress responses [18] by phosphorylating key substrates; such as nuclear factor kappa-light-chain-enhancer of activated B cells (NF-κB), which is involved in inflammation, oxidative stress, and neuronal dysfunction [19,20]. Neflamapimod improved synaptic plasticity and reversed functional memory deficits in a rat model of age-related cognitive decline [17]. In a Phase IIa clinical trial (ClinicalTrials.gov, NCT04001517) in patients with mild-to-moderate Lewy body Dementia (LBD), neflamapimod significantly improved functional mobility and scoring on the Clinical Dementia Rating Scale assessment compared to a placebo without significant treatment-associated adverse effects (TAEs) [21]. Activation of p38 MAPK may also contribute to cancer-related cognitive impairment (CRCI), e.g., chemobrain and chemo fog, which are characterized by deficits in memory and attention, and is commonly experienced alongside CIPN by cancer survivors [22]. Several oral small molecule inhibitors of p38 MAPK have been tried in clinical trials, but were discontinued due to TAEs and their disease-specific limitations [23,24,25,26]. Therefore, considering the safety profile of neflamapimod, we hypothesized that its repurposing to ameliorate CIPN may also offer the advantage of targeting CRCI as well. As a first step, we evaluated the effectiveness and safety-related aspects of neflamapimod in a preclinical model of CIPN as proof of principle for subsequent translational studies.

Cancer may cause pain, even in the absence of chemotherapy [27]. To differentiate between cancer and cisplatin-induced effects, we used a transgenic mouse model of breast cancer expressing a large T-antigen on a C3 promoter (C3TAg) that exhibits the evolutionary spectrum of human breast cancer [28]. C3TAg mice develop ductal atypia at 8 weeks, tumor initiation at ~12 weeks, palpable tumors and hyperalgesia at ~16 weeks, and invasive carcinoma by ~20 weeks, with survival ending at ~25 weeks [28]. The spaced-out intervals of progression in this model offer the opportunity to examine the effect of cisplatin/neflamapimod without interference from pain hypersensitivity that develops due to cancer [29]. We also used healthy FVB/N mice, the genetic background strain of C3TAg to further differentiate between the response of treatments due to cancer and cisplatin. We show that inhibition of p38 MAPK with neflamapimod ameliorates cisplatin-induced injury to DRG neurons and attenuates pain hypersensitivity.

## 2. Materials and Methods

### 2.1. Mice

We used a transgenic mouse model of mammary carcinoma, which shows the evolutionary spectrum of human infiltrating ductal breast carcinoma and expresses the simian virus 40 T-antigen fusion gene on a rat C3 (1) promoter (C3TAg) [28]. Female C3TAg mice develop invasive mammary carcinoma, and males develop prostate cancer [28]. The majority of female C3TAg mice develop atypical changes in the mammary ductal epithelium at 8 weeks, progressing to mammary intraepithelial neoplasia at 12 weeks and invasive carcinomas at 16 weeks of age [28]. Female FVB/N mice, the genetic background strain of C3TAg without tumors, were used to determine the effect on CIPN without the interference of pain due to cancer progression. Mice were assigned randomly to groups, and all conditions were balanced to avoid confounding variables in a double-blinded fashion. Animal use protocols were reviewed and approved by the VA Long Beach Healthcare System Institutional Animal Care and Use Committee (IACUC, protocol #1618941, Approval Date 25 February 2025).

### 2.2. Treatments In Vivo

Animals were block-randomized to treatment groups of medical grade cisplatin (BluePoint Laboratories, Cork, Ireland) and neflamapimod (Tocris, Bristol, UK) [21,30]. Cisplatin was prepared in 0.9% saline (vehicle) and administered at a dose of 2.3 mg/kg/d. Cisplatin or vehicle was administered intraperitoneally (i.p.) for 2 treatment cycles; each cycle included 5 days of treatment followed by 5 days of rest. Behavioral measures were performed over a period of 35 days to assess the sustained effect on CIPN following treatment discontinuation. Neflamapimod was diluted in 1% *w*/*v* pluronic acid in H_2_O and administered by oral gavage (p.o.) at a dose of 6 mg/kg/d, or 1% *w*/*v* pluronic acid vehicle only, twice daily for 35 days. We treated 4 groups of 3-month-old C3TAg and FVB/N mice as follows: Group I: 0.9% saline (i.p.); Group II: cisplatin (i.p.); Group III: cisplatin (i.p.) + neflamapimod (p.o.); Group IV: neflamapimod (p.o.).

### 2.3. Isolation and Culture of DRG Neurons

Female C3TAg and FVB/N mice were euthanized at 3- and 5-months-old using compressed medical-grade CO_2_. The DRG from all vertebral column levels were removed. Neurons were isolated and cultured as previously described [31,32]. The DRG were transferred into Dulbecco’s phosphate buffered saline (DPBS [pH 7.4]; Thermo Scientific, Waltham, MA, USA) on ice and transferred to ice-cold Hank’s balanced salt solution (HBSS [pH 7–7.4]; Thermo Scientific, Waltham, MA, USA) without Ca^2+^ and Mg^2+^. DRG were then dissociated with papain and collagenase/dispase solution in HBSS for 10 min each at 37 °C, with intermittent centrifugation and resuspension of the cell pellet. To inhibit the proteolytic activity of enzymes, the pellet was washed with complete media (Ham’s F-12 Nutrient Mix [Gibco^TM^, Waltham, MA, USA]) containing 10% fetal bovine serum (FBS, Gibco^TM^, Waltham, MA, USA) and 1% penicillin/streptomycin. Finally, a single-cell suspension of DRG neurons was prepared by constant trituration using a fire-polished glass Pasteur pipette. Neurons were cultured on glass coverslips coated with poly-D-lysine and laminin, placed inside 6-well cell culture plates, and used for experiments after 24 h in culture.

### 2.4. Estimation of Intracellular Reactive Oxygen Species (ROS) in DRG Neurons

To quantify intracellular ROS, we utilized a dichlorodihydrofluorescein diacetate (DCFDA or DCF) cellular ROS assay kit (ab113851, Abcam, Waltham, MA, USA) per the manufacturer’s instructions [33]. DRG neurons were cultured on poly-D-lysine and laminin-coated black/clear bottom 96-well plates for 24 h, and then incubated in DCFDA (20 µM in HBSS) for 45 min at 37 °C; this was followed by timed kinetic readings (0–3 h) after treatment with vehicle (0.9% saline/0.2% dimethyl sulfoxide [DMSO]), 6.6 µM cisplatin, 5 µM neflamapimod, or 6.6 µM cisplatin + 5 µM neflamapimod. A 12-h timepoint treatment was followed by DCFDA dye incubation. Fluorescence was measured at excitation/emission (Ex/Em) = 485/535 nm at 37 °C using a SpectraMax M3 plate reader (Molecular Devices, San Jose, CA, USA). Finally, live DRG neurons were counted in each well using the trypan blue dye exclusion test, and fluorescence was normalized to the number of DRG neurons per condition.

### 2.5. Measurement of Mitochondrial Potential in DRG Neurons

Mitochondrial membrane potential was measured using the tetramethylrhodamine ethyl ester (TMRE) mitochondrial membrane potential assay kit (ab113852, Abcam, Waltham, MA, USA) per the manufacturer’s instructions [34]. DRG neurons were cultured on poly-D-lysine and laminin-coated black/clear bottom 96-well plates and treated with vehicle (0.9% saline/0.2% dimethyl sulfoxide [DMSO]), 6.6 µM cisplatin, 5 µM neflamapimod, or a combination of cisplatin 6.6 µM + 5 µM neflamapimod for 2 h, followed by incubation with TMRE (500 nM) for 20 min. Next, the media was replaced with 100 µL of PBS/0.2% bovine serum albumin (BSA), and fluorescence was measured using a SpectraMax M3 plate fluorescence reader (Molecular Devices, San Jose, CA, USA) at Ex/Em = 549/575 nm at 37 °C. Live DRG neurons were counted in each well using the trypan blue dye exclusion test, and fluorescence was normalized to the number of DRG neurons per condition.

### 2.6. Structural Analysis of DRG Neurons

DRG neurons were treated in vitro with vehicle (0.9% saline/0.2% DMSO), cisplatin 6.6 µM, 5 µM neflamapimod, or a combination of 6.6 µM cisplatin + 5 µM neflamapimod. To investigate whether neflamapimod pretreatment prevents cisplatin-induced morphological changes in DRG neurons, neurons were pretreated with 5 µM neflamapimod for 1 h, followed by co-incubation with 6.6 µM cisplatin. To calculate the percentage of pseudo-unipolar DRG neurons, β3-Tubulin-immunostained DRG neurons were analyzed across 6 randomly selected fields of view. Both pseudo-unipolar neurons and the total β3-Tubulin-positive neurons were counted and expressed as a percentage of total cells.

### 2.7. Analysis of p38 MAPK Phosphorylation and Nuclear Colocalization in DRG Neurons

In the first set of experiments, DRG neurons were treated in vitro with vehicle (0.9% saline/0.2% DMSO), cisplatin (0.5, 2, and 6.6 µM), 5 µM neflamapimod, or a combination of 6.6 µM cisplatin and 5 µM neflamapimod. DRG neurons were pretreated with 5 µM neflamapimod for 1 h, followed by co-incubation with 6.6 µM cisplatin. To assess the in vivo effect on neuronal p38 MAPK activation, we isolated DRG neurons after treatment of mice with cisplatin with/without neflamapimod or vehicle as described above. Nuclear colocalization, expressed as a percentage of total cells, and relative phosphorylated p38 MAPK were measured using ImageJ software (v1.53t, National Institutes of Health, Bethesda, MD, USA) after immunostaining as described below.

### 2.8. Immunostaining and Laser-Scanning Confocal Microscopy (LSCM) of DRG Neurons

DRG neurons were immunolabeled as previously described [35,36]. Primary DRG neurons were fixed with 2% paraformaldehyde (Sigma-Aldrich, St. Louis, MO, USA) for 10 min at room temperature (RT). Fixed cells were permeabilized with ice-chilled 0.1% Triton X-100 (Sigma-Aldrich, St. Louis, MO, USA) for 2 min, blocked with 3% donkey serum in PBS for 30 min, and incubated overnight at 4 °C with rabbit anti-mouse phospho-p38 MAPK antibody (#9211S; Cell Signalling Technology [CST], Danvers, MA, USA; 1:100), or rabbit anti-mouse cleaved caspase 3 antibody (#9661; CST, Danvers, MA, USA; 1:200) or rabbit anti-β3-Tubulin (#5568; CST, Danvers, MA, USA; 1:200) diluted in blocking buffer (3% donkey serum in PBS), followed by secondary antibodies, Cy^TM^3 AffiniPure Donkey Anti-Rabbit IgG (H+L), Amax: 550 Emax: 570 nm (#711-165-152, Jackson ImmunoResearch, West Grove, PA, USA), (1:500 dilution in blocking buffer) for 1 h at RT; and mounted with ProLong^TM^ diamond antifade mounting media (Thermo Fisher Scientific, Waltham, MA, USA) containing nuclear stain 4′, 6-diamidino-2-phenylindole (DAPI). Appropriate controls for immunostaining were also prepared and evaluated in parallel for DRG neurons.

Image acquisition and analysis were performed as previously described [36,37]. Z-stack images of 0.5 µm immunostained DRG neurons were acquired on an LSCM (Zeiss LSM-900, Oberkochen, Baden-Württemberg, Germany).

### 2.9. Hyperalgesia Testing

Mice were tested for mechanical, deep/musculoskeletal, and cold hyperalgesia as previously described [38,39]. Mice were familiarized with the testing room environment and apparatus before behavioral analysis. Mice were first tested for mechanical, then musculoskeletal, and lastly for cold hyperalgesia. An adequate amount of rest was provided in between tests. Hyperalgesia measures were obtained at baseline (BL) and on days 2, 5, 10, 16, 20, 30, and 34.

#### 2.9.1. Mechanical Hyperalgesia Testing

Paw withdrawal frequency (PWF) evoked by a 1.0 g (4.08 mN) calibrated von Frey (Semmes–Weinstein) monofilament (Stoelting Co., Wood Dale, IL, USA) was recorded for 10 repeated applications to the plantar surface of each hind paw for 1–2 s, with a force sufficient to bend the filament. Only vigorous withdrawal behaviors were recorded. PWF values are proportional to mechanical hyperalgesia levels.

#### 2.9.2. Musculoskeletal Hyperalgesia Testing

The tensile force of peak forelimb exertion (grip force) was measured using a computerized grip force meter (SA Maier Co., Milwaukee, WI, USA). Each mouse was held by its tail and gently passed over a wire mesh grid during testing, allowed to grip the wires with only its forepaws and gradually pulled by the tail. Three technical replicates of peak force exertion measurement were performed for each mouse. Grip force measurements were normalized to body weight in grams. Grip force is inversely proportional to musculoskeletal/deep tissue hyperalgesia levels.

#### 2.9.3. Cold Hyperalgesia Testing

Cold hyperalgesia was measured as previously described [38]. Mice were placed on a cold plate maintained at 4 °C (U.G.O. Basile Model 35100, Collegeville, PA, USA). The number of paw withdrawals over a 2-min period was recorded as paw withdrawal frequency (PWF). A higher PWF indicates greater cold hypersensitivity. Measurements for PWF were taken once, as cancer-bearing mice are highly sensitive to cold and may not survive excessive cold exposure during repeated tests.

### 2.10. Statistical Analysis

Data analysis was performed using Prism 10.1.324 software (GraphPad, San Diego, CA, USA). One-way or two-way repeated measures ANOVA, followed by Tukey’s post hoc multiple comparisons test, were used to assess within-group differences. A significance threshold of *p* < 0.05 was applied. Additionally, cross-sectional and trend differences in hyperalgesia were evaluated using generalized estimating equation (GEE) models with an assumed autoregressive correlation structure for each outcome, accounting for the repeated nature of the data. We adjusted for baseline outcome, treatment group, log days since baseline, and interaction between log days and treatment group. We did not adjust for the measurement batch as there was little evidence to suggest batch effects on pain outcomes. To account for multiple comparisons, trend-related *p*-values were adjusted using the Benjamini–Hochberg procedure. Data are presented as mean ± SEM.

## 3. Results

### 3.1. In Vitro, Incubation of DRG Neurons with Neflamapimod Inhibits Cisplatin-Induced Phosphorylation and Nuclear Translocation of p38 MAPK

**Figure 1:**
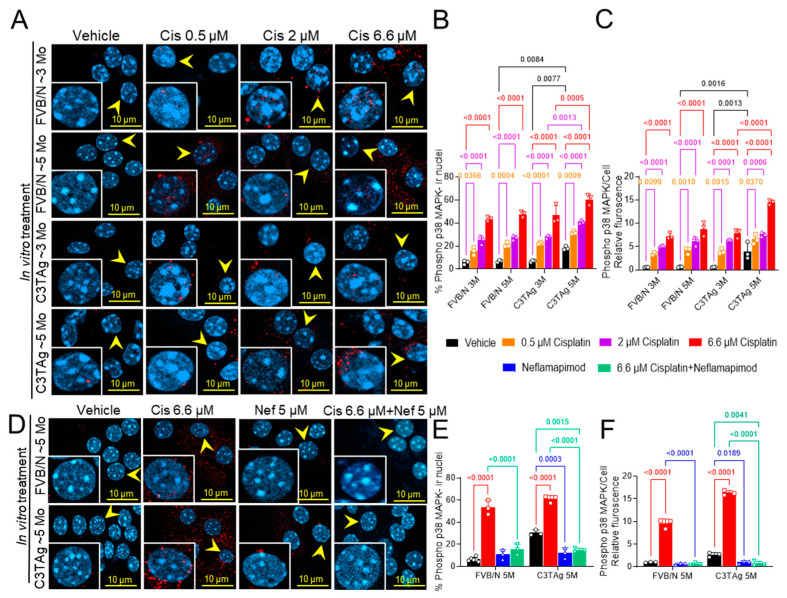
Cisplatin-induced p38 MAPK activation in DRG neurons. (**A**–**C**). Effect of age, genotype, and dose on p38 MAPK phosphorylation and nuclear translocation in the cisplatin-treated DRG neurons of FVB/N and C3TAg female mice. (**D**–**F**). Neflamapimod 5 µM inhibits cisplatin-induced p38 MAPK phosphorylation and nuclear translocation in primary DRG neurons isolated from female FVB/N and C3TAg mice. Images of primary DRG neurons in vitro show co-expression of phospho-p38 MAPK-immunoreactivity (ir, red) and cell nuclei (DAPI, cyan). The yellow arrow indicates phospho-p38 MAPK-ir nuclei. Confocal microscope 63×/NA 1.4 oil was used to capture DRG neurons from 3–6 mice per group. The percentage of phospho-p38 MAPK-ir nuclei and fluorescence was averaged from 6 random fields per group. Data are shown as the mean ± SEM and analyzed with 2-way ANOVA and Tukey’s multiple comparisons test. Abbreviations: DRG, dorsal root ganglia; ir, immunoreactivity; p38 MAPK, P38 mitogen-activated protein kinases; Phospho, phosphorylated.

We investigated if cancer progression led to changes in the DRG neurons that influence cisplatin-induced activation of p38 MAPK by examining the DRG neurons isolated from untreated ~3- and ~5-month-old mice (in vitro study; Figure 1A–C). We examined the dose response of cisplatin in DRG neurons isolated from 3- and 5-month-old FVB/N and C3TAg mice. We found that DRG neurons from ~5-month-old C3TAg mice, with high tumor burden and metastasis, had significantly higher phospho-p38 MAPK nuclear colocalization vs. age-matched FVB/N controls (*p* < 0.05, and *p* < 0.001, respectively), and vs. ~3-month-old C3TAg mice (*p* < 0.01, and *p* < 0.001, respectively). Further, cisplatin induced significant phospho-p38 MAPK nuclear colocalization and increased relative fluorescence in DRG neurons from ~5- and ~3-month-old FVB/N and C3TAg mice vs. respective vehicle-treated groups (*p* < 0.05, and *p* < 0.05, respectively) in a dose-dependent manner for each group. DRG neurons from ~5-month-old C3TAg were more susceptible to cisplatin-induced dose-dependent increase of phospho-p38 MAPK nuclear colocalization and relative fluorescence vs. ~3-month-old C3TAg; thus, suggesting that cancer progression increases the susceptibility of DRG neurons to cisplatin-induced p38 MAPK activation.

Next, we examined the efficacy of neflamapimod, a selective p38α MAPK pharmacological inhibitor, in preventing cisplatin-induced activation of phospho-p38 MAPK in the DRG neurons isolated from ~5-month-old FVB/N and C3TAg mice (Figure 1D–F). Neflamapimod (5 µM) pretreatment significantly reduced cisplatin-induced phospho-p38 MAPK nuclear colocalization and relative fluorescence in FVB/N and C3TAg DRG neurons compared to cisplatin alone (*p* < 0.001 and *p* < 0.001, respectively). Further, 5 µM neflamapimod alone significantly reduced cancer-induced phospho-p38 MAPK nuclear colocalization and relative fluorescence in DRG neurons isolated from ~5-month-old C3TAg vs. vehicle (*p* < 0.01).

### 3.2. Neflamapimod Inhibits Cisplatin-Induced Decrease in Oxidative Stress in Isolated DRG Neurons

**Figure 2:**
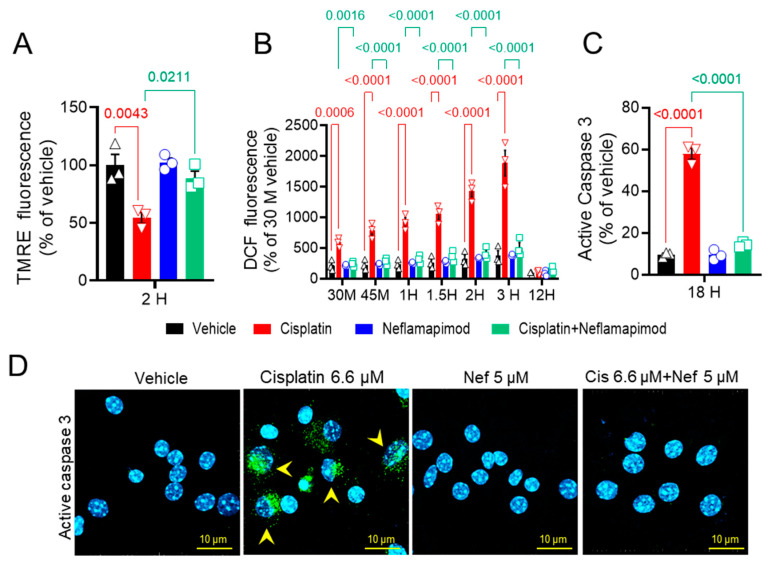
In vitro neflamapimod inhibits cisplatin-induced oxidative stress and active caspase 3 in DRG neurons of C3TAg mice. DRG neurons isolated from 3-month-old female C3TAg mice were treated in vitro with vehicle 0.9% saline/0.2% DMSO, cisplatin 6.6 µM, neflamapimod 5 µM, or a combination of cisplatin 6.6 µM + neflamapimod 5 µM. (**A**) Mitochondrial potential (ΔΨM), 2-h post-treatment. (**B**). Reactive oxygen species (ROS), 30 and 45 min and 1-, 1.5-, 2-, 3-, and 12-h post-treatment. (**C** and **D**) Active caspase 3, 18-h post treatment. Confocal microscope magnification: 63×/1.4 oil. Data are shown as the mean ± SEM analyzed with one and 2-way ANOVA and Tukey’s multiple comparisons test. Abbreviations: DCF, dichlorofluorescein; DMSO, dimethyl sulfoxide; DRG, dorsal root ganglia; TMRE, tetramethyl rhodamine ethyl ester.

We next assessed the change (Δ) in mitochondrial membrane potential (ΨM) in primary DRG neurons isolated from tumor-bearing C3TAg mice (Figure 2A). We found that 6.6 µM cisplatin significantly reduced ΔΨM after 2 h vs. vehicle (*p* < 0.01), and co-incubation with 5 µM neflamapimod + 6.6 µM cisplatin significantly prevented the reduction of ΔΨM vs. 6.6 µM cisplatin treatment (*p* < 0.05). No significant changes in ΔΨM were observed in the 5 µM neflamapimod alone and the 6.6 µM cisplatin + 5 µM neflamapimod-treated groups vs. vehicle.

### 3.3. Neflamapimod Protects DRG Neurons from Cisplatin-Induced Reactive Oxygen Species (ROS) Bursts

Mitochondrial integrity is affected by ROS. Therefore, we examined the effect of neflamapimod on intracellular ROS induction following cisplatin exposure in primary DRG neurons using DCFDA (Figure 2B). We observed a time-dependent increase in ROS levels in DRG neurons following 6.6 µM cisplatin treatment: at 0.5 h (*p* < 0.001), 0.75 h (*p* < 0.0001), 1 h (*p* < 0.0001), 1.5 h (*p* < 0.0001), 2 h (*p* < 0.001), and 3 h (maximal change, *p* < 0.0001) vs. vehicle at the matching timepoints. The DRG neurons pretreated for 1 h with 5 µM neflamapimod prior to 6.6 µM cisplatin exposure showed significant reduction in ROS levels at 0.5 h (*p* < 0.001), 0.75 h (*p* < 0.0001), 1 h (*p* < 0.0001), 1.5 h (*p* < 0.0001), and 2 h (*p* < 0.0001) compared to 6.6 µM cisplatin treatment alone. No significant change was observed in the 5 µM neflamapimod or 6.6 µM cisplatin + 5 µM neflamapimod-treated groups vs. vehicle. At the 12-h time point after cisplatin treatment with and without neflamapimod, there was no significant difference in ROS levels in the DRG neurons compared to vehicle. It is plausible that cisplatin-induced ROS production underlies alterations in the mitochondrial membrane potential via a p38 MAPK-mediated mechanism, which is inhibited by neflamapimod.

### 3.4. Neflamapimod Inhibits Cisplatin-Induced Activation of Caspase 3 in DRG Neurons

Caspase 3 activation in DRG neurons is associated with the development of neuropathic pain [40]. To further understand the neuroprotective mechanism of neflamapimod in cisplatin-treated DRG neurons, we measured active caspase 3 expression in primary DRG neurons (Figure 2C and D). The green puncta represent immunolabelling for active caspase 3 expression, and cyan depicts the nuclei of DRG neurons. Exposure to 6.6 µM cisplatin (18 h) significantly increased the number of active caspase 3-positive cells vs. vehicle (*p* < 0.0001) in DRG neurons isolated from ~3-month-old C3TAg mice, which was prevented by 1 h of pretreatment with 5 µM neflamapimod (*p* < 0.0001). Further, there was no significant change in the number of active caspase 3-positive DRG neurons in the 5 µM neflamapimod alone treated group vs. vehicle as a percentage of total neurons. Caspase 3 activation contributes to apoptosis, suggesting that neflamapimod prevents cisplatin-induced neuronal damage.

### 3.5. Neflamapimod Inhibits Cisplatin-Induced Morphological Alterations in DRG Neurons

**Figure 3:**
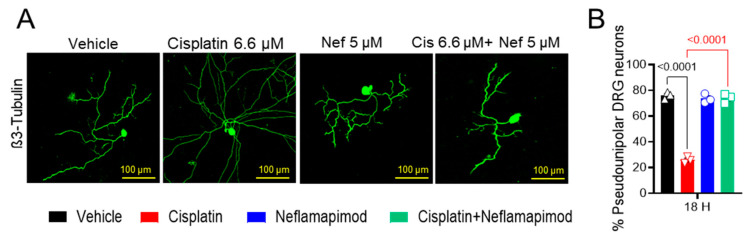
Neflamapimod inhibits cisplatin-induced morphological change in DRG neurons. DRG neurons isolated from ~3-month-old female C3TAg mice were treated in vitro with vehicle 0.9% saline/0.2% DMSO, cisplatin 6.6 µM, neflamapimod 5 µM, or a combination of cisplatin 6.6 µM + neflamapimod 5 µM. (**A**) Representative images showing structural changes in DRG neurons, and (**B**) quantification of pseudounipolar neurons. Confocal microscope magnification: 20×/NA 0.8. Age: ~3 months. Data are shown as the mean ± SEM and analyzed with one-way ANOVA and Tukey’s multiple comparisons test. Abbreviations: DMSO, dimethyl sulfoxide; DRG, dorsal root ganglia; Nef, neflamapimod.

We found that a high percentage of DRG neurons isolated from tumor-free ~3-month-old C3TAg mice displayed pseudounipolar morphology in primary cell culture (Figure 3A,B). 6.6 µM cisplatin treatment for 18 h distorted the pseudounipolarity of DRG neurons compared to vehicle treatment (*p* < 0.0001), and multiple neurites emerged from the soma following exposure to cisplatin. The 1-h pretreatment with 5 µM neflamapimod prior to cisplatin exposure helped to maintain pseudounipolarity of the DRG neurons compared to cisplatin treatment alone (*p* < 0.0001). Thus, cisplatin leads to neuronal damage resulting in sprouting, a sign of neuropathic injury.

### 3.6. In Vivo Neflamapimod Treatment in Tumor-Bearing C3TAg and Control FVB/N Mice Reduces Cisplatin-Induced Phospho-p38 MAPK Phosphorylation

**Figure 4:**
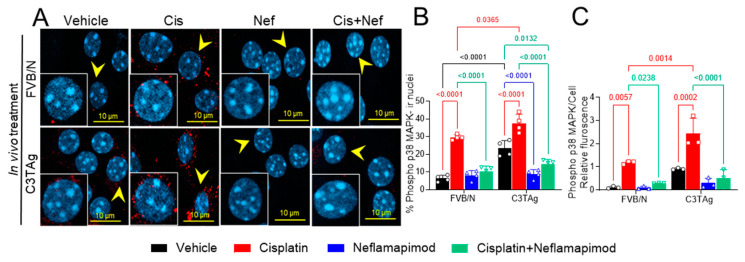
In vivo neflamapimod inhibits cisplatin-induced phopho-p38 MAPK activation and colocalization in nuclei of DRG neurons. C3TAg and FVB/N mice were treated with vehicle, neflamapimod, and cisplatin ± neflamapimod as described under the Methods. DRG neurons were isolated from all groups of mice after treatments. (**A**) Images of primary DRG neurons show the co-expression of phospho-p38 MAPK-immunoreactivity (ir, red) and cell nuclei (DAPI, cyan). Yellow arrows indicate the phospho-p38 MAPK-ir nuclei. Confocal microscope magnification: 63×/1.4 oil. Z-stacks of 0.5 µm images from 6 fields of view were captured for each condition, from DRG neurons obtained from 4–6 mice per condition. (**B**,**C**) The percentage of phospho-p38 MAPK-ir nuclei and fluorescence was averaged from 6 randomly selected fields per group. Data are shown as the mean ± SEM analyzed with 2-way ANOVA and Tukey’s multiple comparisons test. Abbreviations: ir, immunoreactivity; p38 MAPK, P38 mitogen-activated protein kinase; Phospho, phosphorylated.

**Figure 5:**
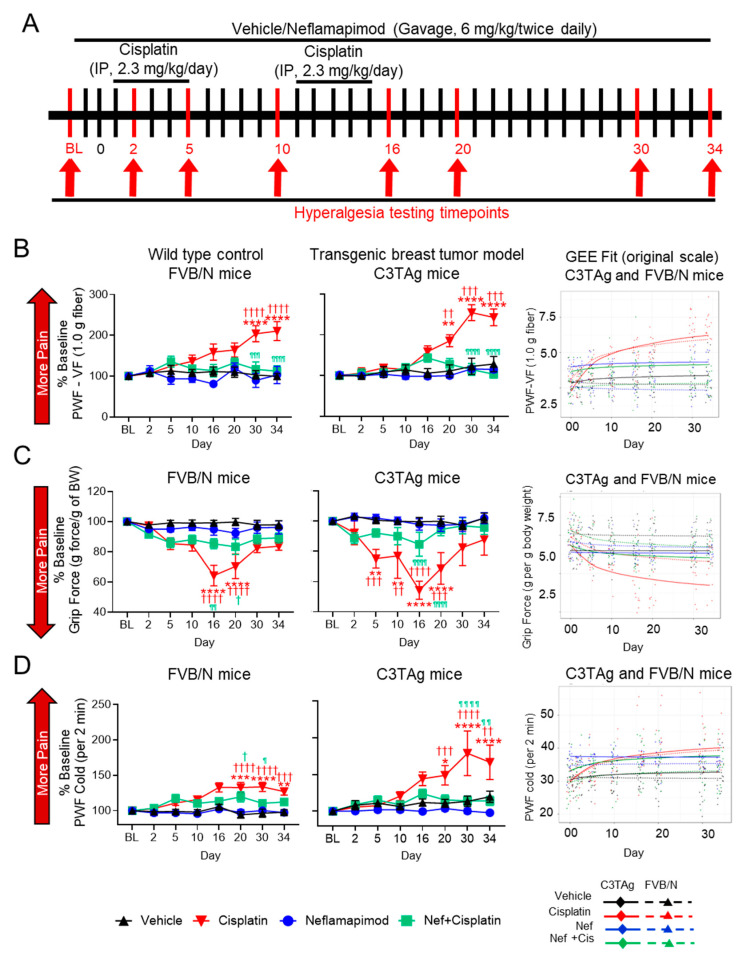
Neflamapimod ameliorates cisplatin-induced hyperalgesia in FVB/N control and C3TAg mice. (**A**) Female C3TAg and FVB/N mice, ~3-month-old, were administered vehicle or cisplatin (2.3 mg/kg body weight/day, i.p.) for 2 cycles (cycle 1: day 1–5, break for 5 days, cycle 2: day 10–15). Neflamapimod (Nef) was administered (6 mg/kg body weight) 2 times daily by oral gavage. Dosing was started 2 days prior to cisplatin treatment and continued until day 34. Hyperalgesia measures were obtained at baseline (BL) and on days 2, 5, 10, 16, 20, 30, and 34 after treatment. (**B**) Mechanical hyperalgesia; (**C**) grip force; (**D**) cold hyperalgesia. FVB/N: Vehicle (n = 10), cisplatin (n = 8), neflamapimod (n = 6), neflamapimod + cisplatin (n = 10); C3TAg: vehicle (n = 9), cisplatin (n = 6), neflamapimod (n = 6), neflamapimod + cisplatin (n = 6). Analyzed with two-way ANOVA with Tukey’s multiple comparisons test. Symbols: * significance compared to BL; † significance compared to vehicle at the matching time point; ^¶^ significance compared cisplatin at the matching time point; ^*,†,¶^ *p* < 0.05; ^**,††,¶¶^ *p* < 0.01; ^***,†††,¶¶¶^ *p* < 0.001; ^****,††††,¶¶¶¶^ *p* < 0.0001. Abbreviations: BL, baseline; BW, body weight; GEE, generalized estimating equations; PWF, paw withdrawal frequency; VF, von Frey.

Signaling of p38 MAPK in the PNS contributes to chronic pain and may underlie hyperalgesia features in a cisplatin-induced CIPN rodent model [9]. The DRG neurons were isolated from tumor-bearing C3TAg and wild-type control FVB/N mice and evaluated for phospho-p38 MAPK (Figure 4A and B) on day 34 following the cisplatin ± neflamapimod treatment regimen described in Figure 5A. We observed that cisplatin treatment led to a significant increase in phospho-p38 MAPK nuclear translocation and relative fluorescence in DRG neurons from FVB/N (*p* < 0.0001, *p* < 0.01, respectively) and C3TAg mice (*p* < 0.0001, *p* < 0.001, respectively) vs. vehicle. Both pretreatment with neflamapimod and neflamapimod cotreatment with cisplatin reduced cisplatin-induced phospho-p38 MAPK nuclear colocalization and relative fluorescence in DRG neurons isolated from FVB/N (*p* < 0.0001, *p* < 0.05, respectively) and C3TAg mice (*p* < 0.0001, *p* < 0.0001, respectively). Additionally, DRG neurons isolated from the C3TAg vehicle-treated mice showed higher phospho-p38 MAPK nuclear colocalization (*p* < 0.0001) vs. the FVB/N vehicle-treated mice. The cisplatin-treated C3TAg mice showed a higher phospho-p38 MAPK nuclear colocalization level and relative fluorescence vs. cisplatin-treated FVB/N control mice (*p* < 0.05, *p* < 0.001, respectively).

These observations suggest that cancer progression leads to sensitization of p38 MAPK activation which is influenced by cisplatin and neflamapimod in vivo (Figure 4), similar to the cisplatin-induced activation of p38 MAPK and inhibition with neflamapimod in neurons treated in vitro after isolation from DRG in Figure 1. Cisplatin treatment in vivo may have a greater impact on mechanisms underlying hyperalgesia, perhaps due to an underlying sensitization caused by cancer.

### 3.7. Neflamapimod Ameliorates Cisplatin-Induced Hyperalgesia in Transgenic Breast Cancer C3TAg Mice and Control FVB/N Mice

Neflamapimod ameliorates cisplatin-induced hyperalgesia in C3TAg mice and FVB/N control mice, reducing mechanical, musculoskeletal, and cold hyperalgesia associated with CIPN (Figure 5A–D).

#### 3.7.1. Mechanical Hyperalgesia

Activation of the p38 MAPK signaling pathway contributes to chronic pain and may underlie mechanical hyperalgesia in a CIPN model [41]. Cisplatin treatment (2.3 mg/kg/day for 2 cycles) resulted in mechanical hyperalgesia when compared to vehicle-treated transgenic C3TAg breast cancer mice and FVB/N control mice. In both C3TAg and FVB/N mice, cisplatin induced a progressive increase in PWF vs. baseline in response to von Frey monofilament application, indicating the development of mechanical hyperalgesia (Figure 5B). This increase became significant by day 20 (C3TAg: *p* < 0.01 vs. vehicle at the matching time point; *p* < 0.01 vs. baseline) and intensified by day 30 (C3TAg: *p* < 0.001 vs. vehicle at the matching time point; *p* < 0.0001 vs. baseline; FVB/N: *p* < 0.0001 vs. vehicle at the matching time point and baseline) and persisted through day 34 (C3TAg: *p* < 0.001 vs. vehicle at the matching time point; *p* < 0.0001 vs. baseline; FVB/N: *p* < 0.0001 vs. vehicle at the matching time point and baseline). C3TAg mice exhibited a greater increase in mechanical hyperalgesia compared to FVB/N mice, suggesting heightened sensitivity to cisplatin-induced neuropathy. Co-administration of neflamapimod with cisplatin significantly reduced measures of mechanical hyperalgesia in C3TAg and FVB/N mice, with a significant reduction observed at day 30 (C3TAg: *p* < 0.0001 vs. cisplatin at the matching time point; FVB/N: *p* < 0.001 vs. cisplatin at the matching time point) and day 34 (C3TAg: *p* < 0.0001 vs. cisplatin at the matching time point; FVB/N: *p* < 0.0001 vs. cisplatin at the matching time point).

To further validate these findings and account for the longitudinal nature of the data, generalized estimating equation (GEE) models were applied to assess both cross-sectional differences and trend effects over time. GEE analysis confirmed that cisplatin treatment resulted in sustained mechanical hyperalgesia, persisting beyond treatment cessation (day 16; C3TAg CI: (−2.65, −1.68), *p* < 0.0001; FVB/N CI: (−2.81, −1.82), *p* < 0.0001) and post-treatment recovery (day 34; C3TAg CI: (−3.53, −2.25) *p* < 0.0001; FVB/N CI: (−3.6, −2.32), *p* < 0.0001), which are suggestive of CIPN. Neflamapimod (Nef, 6 mg/kg twice daily) significantly attenuated mechanical hyperalgesia arising from CIPN following two cycles of cisplatin treatment in female, C3TAg, and FVB/N mice following cessation of cisplatin (day 16; C3TAg CI: (1.35, 2.16), *p* < 0.0001; FVB/N CI: (1.48, 2.49), *p* < 0.0001) and post-treatment recovery (day 34; C3TAg CI: (1.97, 3.02) *p* < 0.0001; FVB/N CI: (1.85, 3.17), *p* < 0.0001). The day-by-day trajectory of mechanical hyperalgesia was also significantly attenuated by neflamapimod (trend; C3TAg CI: (0.82, 1.23) *p* < 0.0001; FVB/N CI: (0.49, 0.95), *p* < 0.0001).

#### 3.7.2. Musculoskeletal/Deep Tissue Hyperalgesia

Difficult-to-treat, spontaneous musculoskeletal hyperalgesia is a known consequence of cancer progression and cisplatin-induced peripheral neuropathy (CIPN) [42]. Cisplatin treatment (2.3 mg/kg/day for two cycles) resulted in significant neuromuscular dysfunction in FVB/N and C3TAg mice, as indicated by a progressive reduction in grip strength (Figure 5C). This decline became significant by day 5 (C3TAg: *p* < 0.001 vs. vehicle at the matching time point; *p* < 0.01 vs. baseline) and worsened by day 10 (C3TAg: *p* < 0.01 vs. vehicle at the matching time point; *p* < 0.01 vs. baseline), reaching its most severe levels by day 16 (C3TAg: *p* < 0.0001 vs. vehicle at the matching time point and baseline; FVB/N: *p* < 0.0001 vs. vehicle at the matching time point and baseline) and day 20 (C3TAg: *p* < 0.0001 vs. vehicle at the matching time point; *p* < 0.001 vs. baseline; FVB/N: *p* < 0.0001 vs. vehicle at the matching time point and baseline). Co-administration of neflamapimod with cisplatin significantly attenuated this decline in both strains, with improvements observed by day 16 (C3TAg: *p* < 0.0001 vs. cisplatin at the matching time point; FVB/N: *p* < 0.01 vs. cisplatin at the matching time point) and day 20 (C3TAg: *p* < 0.0001 vs. cisplatin at the matching time point; FVB/N: *p* < 0.05 vs. vehicle at the matching time point).

Analysis of cross-sectional and trend differences revealed that cisplatin treatment significantly reduced grip strength in female C3TAg and FVB/N mice compared to vehicle treatment. This decline was evident following treatment cessation (day 16; C3TAg CI: (1.05, 2.17), *p* < 0.0001; FVB/N CI: (0.62, 1.45), *p* < 0.0001) and post-treatment recovery (day 34; C3TAg CI: [1.32, 2.78] *p* < 0.0001; FVB/N CI: [0.76, 1.79], *p* < 0.0001), which are suggestive of musculoskeletal hyperalgesia. Neflamapimod significantly improved grip force in female C3TAg mice following cessation of cisplatin (day 16; C3TAg CI: (−1.81, −0.49), *p* < 0.01), post-treatment recovery (day 34; C3TAg CI: (−2.34, −0.58), *p* < 0.001), and day-by-day trend (trend; C3TAg CI: (−0.65, −0.07) *p* < 0.0001), suggesting a sustained improvement in musculoskeletal hyperalgesia. These findings reinforce the role of cisplatin-induced neuromuscular dysfunction in musculoskeletal hyperalgesia while highlighting neflamapimod’s potential to restore motor function and grip force over time.

#### 3.7.3. Cold Hyperalgesia

Cold-induced hyperalgesia is a common feature of CIPN, associated with altered sensory processing and neuropathic pain [43]. Cisplatin treatment resulted in significant cold hyperalgesia in FVB/N and C3TAg mice, as indicated by a progressive increase in PWF to cold stimuli (Figure 5D). This increase became significant by day 20 (C3TAg: *p* < 0.001 vs. vehicle at the matching time point; *p* < 0.01 vs. baseline; FVB/N: *p* < 0.0001 vs. vehicle at the matching time point and baseline) and worsened by day 30 (C3TAg: *p* < 0.0001 vs. vehicle at the matching time point and baseline; FVB/N: *p* < 0.0001 vs. vehicle at the matching time point and baseline) and day 34 (C3TAg: *p* < 0.01 vs. vehicle at the matching time point; *p* < 0.0001 vs. baseline; FVB/N: *p* < 0.01 vs. vehicle at the matching time point; *p* < 0.001 vs. baseline). Co-administration of neflamapimod with cisplatin significantly prevented cold hyperalgesia in C3TAg and FVB/N mice, with a significant reduction in PWF observed by day 20 (FVB/N: *p* < 0.05 vs. vehicle), day 30 (C3TAg: *p* < 0.0001 vs. cisplatin at the matching time point; FVB/N: *p* < 0.01 vs. cisplatin at the matching time point) and day 34 (C3TAg: *p* < 0.0001 vs. cisplatin at the matching time point; FVB/N: *p* < 0.0001 vs. cisplatin at the matching time point).

Analysis of cross-sectional and trend differences revealed that cisplatin treatment significantly increased PWF to cold stimuli in female C3TAg and FVB/N mice compared to vehicle-treatment. This increase was evident following treatment cessation (day 16; C3TAg CI: [−9.36, −2.87], *p* < 0.001; FVB/N CI: [−11.37, −4.44], *p* < 0.0001) and persisted through post-treatment recovery (day 34; C3TAg CI: [−12.01, −3.57], *p* < 0.001; FVB/N CI: [−14.33, −5.79], *p* < 0.0001), which is suggestive of CIPN-related cold hyperalgesia. Co-administration of neflamapimod with cisplatin significantly reduced cold PWF in C3TAg mice, with improvements observed at treatment cessation (day 16; C3TAg CI: [2.32, 7.07], *p* < 0.001; FVB/N CI: [1.34, 7.5], *p* < 0.01) and post-treatment recovery (day 34; C3TAg CI: [0.16, 8.71], *p* < 0.05; FVB/N CI: [1.9, 9.51], *p* < 0.001).

## 4. Discussion

**Figure 6:**
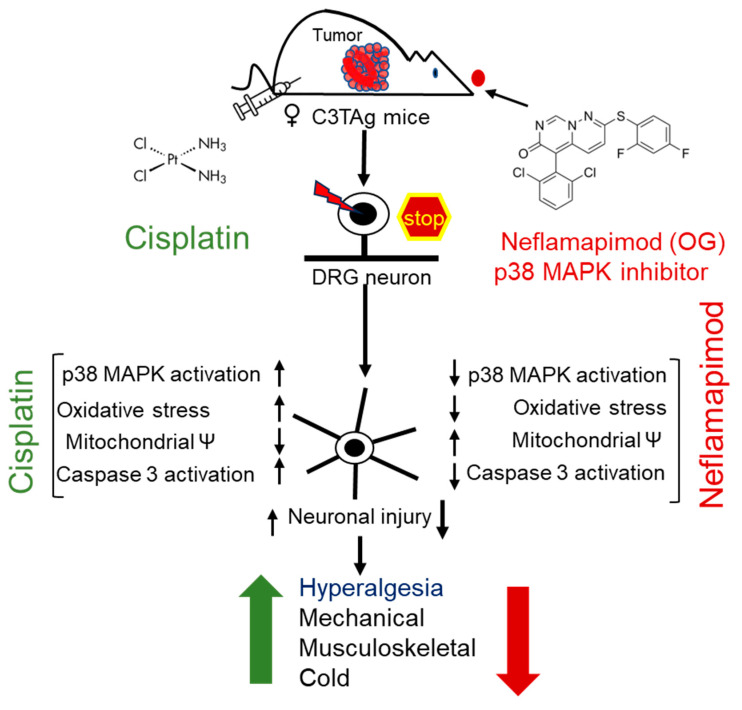
Cisplatin-induced hyperalgesia is mitigated by a p38 MAPK inhibitor neflamapimod. Cisplatin treatment activates p38 MAPK in DRG neurons of C3TAg mice with mammary tumors. Neflamapimod, a p38α MAPK inhibitor, inhibits this activation and reduces cisplatin-induced oxidative stress, mitochondrial dysfunction, and apoptosis. It preserved neuronal integrity and axonal structure, while ameliorating cisplatin-induced hyperalgesia. The green upward arrow represents an increase in hyperalgesia following cisplatin treatment, while the red downward arrow indicates a reduction with neflamapimod co-treatment. Abbreviations: DRG, dorsal root ganglia; OG, oral gavage; p38 MAPK, p38 Mitogen-activated protein kinase.

In this proof of principle study, we demonstrated the therapeutic potential of neflamapimod to ameliorate CIPN in a translational murine model with an evolutionary spectrum of breast cancer (Figure 6). Cisplatin binds to both genomic and mitochondrial DNA, forming platinum-DNA adducts [44]. In DRG neurons, this impairs mitochondrial function and causes electron leakage in the electron transport chain (ETC), leading to excessive ROS production [12]. Notably, in DRG neurons, mitochondrial dysfunction and oxidative stress are underlying mechanisms involved in CIPN [12,13]. In primary DRG neurons, cisplatin depletes mitochondrial DNA, induces loss of mitochondrial membrane potential, elevates ROS levels, and activates caspases, leading to axonal degeneration [42,45]. Therefore, we focused on mitochondrial mechanisms and neuroprotective effects of neflamapimod mediated by inhibition of p38 MAPK.

In our study, we found that cisplatin treatment in transgenic breast cancer C3TAg mice and FVB/N control mice resulted in early and prolonged mechanical, musculoskeletal/deep tissue, and cold hyperalgesia, accompanied by increased damage to DRG neurons, consistent with previous CIPN findings. In several rodent studies, p38 MAPK activation is interlinked with axonal damage and morphological changes in DRG neurons [46]. Axonal damage can lead to loss of pseudo-unipolarity of DRG neurons, a feature characterized by multiple neuritic growths from the soma [47]. Sprouting of sensory neurons in the DRG is a key feature of nerve injury and contributes to neuropathic pain [48].

We found that cisplatin directly activated p38 MAPK in DRG neurons isolated from both transgenic breast cancer and FVB/N control mice in a dose-dependent manner, with significantly higher activation observed in tumor-bearing C3TAg (~5-month-old) mice. Tumor necrosis factor (TNF)-α is a known activator of p38 MAPK, and elevated TNF-α in the bloodstream associated with cancer is known to activate the p38 MAPK signaling pathway and cause associated neuropathic pain [49]. Our findings suggest that cisplatin administration in tumor-bearing C3TAg mice worsens hyperalgesia by further increasing p38 MAPK activation and causing damage to the PNS. Low-dose cisplatin causes injury to hippocampal neurons which may underlie CRCI [50]. It is likely that neflamapimod may also protect against injury to hippocampal neurons induced by cisplatin. In fact, the cholinergic neuronal degeneration observed in Rab-5 overexpressing mice was inhibited by neflamapimod via inhibition of p38α MAPK phosphorylation and reduced levels of its downstream substrates MK2 and MNK1 [21]. It is noteworthy that, in addition to being activated by upstream MAP kinase kinases, p38α MAPK can autoactivate itself; thus, inhibition of p38α MAPK is critical in treating CIPN [51].

Neflamapimod is a potent and selective p38α MAPK inhibitor with a high safety profile at extremely high doses in clinical studies involving hundreds of patients [17,52,53]. We show that neflamapimod prevents cisplatin-mediated activation of the p38 MAPK in the DRG neurons of age-matched tumor-bearing C3TAg mice and FVB/N control mice, both in vitro and in vivo. Neflamapimod treatment markedly reversed cisplatin-induced neuropathic pain by reducing paw withdrawal frequency following mechanical and cold stimuli, and improved grip force. Collectively, these findings are consistent with reduced thermal hypersensitivity and musculoskeletal hyperalgesia, respectively. Further, we found that the levels of phospho-p38 MAPK in DRG neurons corresponded with hyperalgesia in tumor-bearing C3TAg and FVB/N mice. Analysis of body weight, organ weights, and hematology profile did not show any adverse effects of neflamapimod in both FVB/N or C3TAg mice after treatment. Thus, neflamapimod’s therapeutic effect on CIPN appears to be safe.

Downstream activation of caspase 3 is a sign of neuronal apoptosis, while its upregulation may be involved in axonal damage-induced neurite sprouting [54,55]. Inhibition of the phospho-p38 MAPK in the DRG neurons of C3TAg mice by neflamapimod prevented ΨM loss, bursts of ROS, and axonal damage, indicating its neuroprotective properties. Neflamapimod prevents cisplatin-induced oxidative stress, which is likely dependent on phosphorylation of the p38 MAPK pathway, thus attenuating neuronal injury. Overall, we show that neflamapimod protects against cisplatin-induced neurotoxicity in DRG neurons. Our data complement the mechanisms of activation of sodium channels by p38 MAPK phosphorylation in DRG neurons, which may contribute to neuropathic pain [56]. SB203580, an inhibitor of p38 MAPK, significantly inhibited the TNF-α-mediated Nav1.7 (a known contributor to pain and neuronal excitability) current density in the DRG-derived ND7/23 cell line [57,58,59]. Furthermore, proinflammatory cytokine interleukin-1 beta (IL-1ß) signals through p38 MAPK and inhibits brain-derived neurotrophic factor (BDNF), leading to modulation of neuronal plasticity [60]. IL-1ß and p38 MAPK isoforms gamma and delta are involved in the development of T-cells, thus selective inhibition of p38α MAPK is not a concern [52,53,61]. In several clinical studies on hundreds of patients, neflamapimod has not led to an increase in infections or immunosuppression [21,52,62]. Thus, neflamapimod shows a neuroprotective profile without TAEs.

p38 MAPK may also contribute to chemotherapy-induced cognitive and behavioral deficits [63]; inhibition of the p38 MAPK/MK2 pathway attenuates breast cancer progression, metastasis, and chemotherapy-induced bone loss in an orthotopic mouse model of breast cancer [64]. Collectively, our data on the role of P38 MAPK in CIPN and therapeutic benefit of targeting p38 MAPK activation with neflamapimod provides a proof of principle for clinical translation pending evaluation of its safety and effectiveness in persons with cancer.

## 5. Conclusions

p38 MAPK is known to contribute to neuropathic pain including cold allodynia and hyperalgesia, which are symptoms observed in CIPN [65]. Our observations that cisplatin can directly activate phospho-p38 MAPK in DRG neurons are consistent with earlier observations. There is no established FDA-approved treatment to address CIPN and its underlying pathophysiology. Our findings demonstrate the neuroprotective effectiveness of neflamapimod in a mouse model of CIPN and identify phospho-p38 MAPK as a key therapeutic target. Neflamapimod has been utilized in many clinical trials for neuroprotective effects and cognitive function, including Alzheimer’s disease and LBD [66,67]. Even at extremely high doses, no significant TAEs have been reported for neflamapimod in several clinical studies; therefore, we are cautiously optimistic that our preclinical findings provide a proof of principle for the development of neflamapimod as a mitigator of CIPN following clinical trials for safety and efficacy.

## Data Availability

All data are included in the manuscript.

## Abbreviations

The following abbreviations are used in this manuscript:

BL	baseline
BW	body weight
CIPN	Chemotherapy-induced peripheral neuropathy
DAPI	4′, 6-diamidino-2-phenylindole
DCF	2′,7′-Dichlorofluorescein
DCFDA	2′,7′-Dichlorodihydrofluorescein diacetate
DRG	dorsal root ganglia
Ex/Em	excitation/emission
HBSS	Hank’s balanced salt solution
LSCM	laser scanning confocal microscopy
p38 MAPK	p38 mitogen-activated protein kinase
PBS	phosphate-buffered saline
PNS	peripheral nervous system
PWF	paw withdrawal frequency
ROS	reactive oxygen species
RT	room temperature
TMRE	tetramethylrhodamine
TNF-α	tumor necrosis factor alpha
VF	von Frey
